# Fetal Reduction by Potassium Chloride Infusion in Unruptured Heterotopic Pregnancy: A Comprehensive Review

**DOI:** 10.7759/cureus.53618

**Published:** 2024-02-05

**Authors:** Manjusha Agrawal, Lucky Srivani Reddy, Drashti Patel, Garapati Jyotsna, Archan Patel

**Affiliations:** 1 Obstetrics and Gynecology, Jawaharlal Nehru Medical College, Datta Meghe Institute of Higher Education and Research, Wardha, IND

**Keywords:** clinical outcomes, risk mitigation, reproductive medicine, unruptured heterotopic pregnancy, potassium chloride infusion, fetal reduction

## Abstract

This comprehensive review explores the practice of fetal reduction through potassium chloride infusion in unruptured heterotopic pregnancies. Heterotopic pregnancies, characterized by the simultaneous occurrence of intrauterine and extrauterine gestations, present unique challenges in reproductive medicine. The review defines fetal reduction and underscores its significance in mitigating risks associated with heterotopic pregnancies, including the threat of rupture, maternal morbidity, and adverse outcomes. The analysis encompasses the background, methods, efficacy, ethical considerations, and future directions related to the procedure. Findings highlight the efficacy and safety of potassium chloride infusion, emphasizing the importance of proper patient selection and counseling. Implications for clinical practice underscore the procedure's viability in specific cases where the benefits outweigh the associated risks. The review concludes with recommendations for future studies, encouraging further research on procedural techniques, alternative methods, and the psychosocial impact on patients. This work is a foundation for advancing the management of unruptured heterotopic pregnancies, providing insights for clinicians and researchers to improve clinical outcomes and patient care.

## Introduction and background

Fetal reduction refers to a medical procedure aimed at reducing the number of fetuses in multiple pregnancies, typically to improve the chances of a healthy outcome for the remaining fetuses. This intervention is particularly relevant in cases of heterotopic pregnancy (HP), where there is a simultaneous occurrence of intrauterine and extrauterine pregnancies. Fetal reduction involves the selective reduction of one or more embryos to mitigate the risks associated with a higher-order multiple gestation [1]. Unruptured HP poses a unique challenge in reproductive medicine, as it involves the coexistence of embryos both within the uterus and in an ectopic location, such as the fallopian tube. The significance of fetal reduction in this context lies in its potential to address the increased risks and complications associated with HP, including the threat of rupture, maternal morbidity, and adverse outcomes for both intrauterine and extrauterine gestations [2].

The purpose of this comprehensive review is to critically examine the practice of fetal reduction using potassium chloride (KCl) infusion in cases of unruptured HP. By synthesizing existing literature, we aim to provide a thorough understanding of the background, methods, efficacy, ethical considerations, and future directions related to this specific approach. This review aims to contribute valuable insights to the medical community, guiding clinicians in decision-making and offering directions for further research and improvement in the management of unruptured HP through fetal reduction.

## Review

Background

Overview of HP

HP is an uncommon medical condition characterized by the simultaneous presence of an intrauterine and an ectopic pregnancy. This condition poses a potential threat to life, and its occurrence has escalated due to the growing utilization of ART. The primary risk factors associated with HP mirror those of ectopic pregnancy and encompass a history of ectopic pregnancy, tubal surgery, and pelvic inflammatory disease. Diagnosis typically involves a comprehensive evaluation, combining clinical presentation, pelvic imaging, and sonographic visualization of both intrauterine and extrauterine pregnancies. The management of HP may encompass diverse approaches, such as expectant management, methotrexate (MTX) therapy, surgery, and, in certain instances, ultrasound-guided fetal reduction involving KCl. However, it is essential to note that the use of KCl for fetal reduction carries a considerable risk and requires meticulous consideration. Given the rising incidence of HP, early diagnosis and appropriate management are imperative to ensure optimal outcomes for the patients involved [3-5].

Incidence and Prevalence

The occurrence of spontaneous HP is considered rare, affecting approximately one in 30,000 pregnancies [4]. However, there is a notable trend indicating a rise in incidence attributed to the widespread adoption of ART and ovulation induction. A specific study highlights that the estimated incidence of HP is one in 30,000 in spontaneous pregnancies but significantly higher at one in 3900 when associated with ART or ovulation induction [6]. The increased prevalence of multiple pregnancies resulting from ovulation induction and ART procedures contributes substantially to the elevated occurrence of HP. Consequently, although still infrequent, the incidence of HP may be more pronounced in the context of ART and ovulation induction [4,6,7].

Risks and Complications Associated with HP

Ectopic pregnancy, identified by the presence of a fertilized egg implanting outside the uterus, introduces a heightened risk of severe complications. These complications may include the potential for rupture, hemorrhage, and, in extreme cases, maternal death [8]. Immediate medical attention and intervention are imperative to avert life-threatening consequences associated with this emergent condition. In conjunction with the risks to the ectopic pregnancy itself, the coexistence of an intrauterine pregnancy (IUP) introduces complexities. Shared physiological space may lead to complications for the developing fetus, emphasizing the need for meticulous management to ensure the well-being of both pregnancies [8]. Diagnosing HP presents a unique challenge due to symptoms that may overlap with other early pregnancy complications. Accurate diagnosis typically relies on a comprehensive assessment, incorporating physical examination, advanced imaging through ultrasound, and relevant blood tests [8]. Timely and precise diagnosis is critical for initiating appropriate and timely management strategies. The approach to treating HP is contingent on factors such as the location of the ectopic pregnancy and the patient's clinical presentation. Common treatment modalities encompass surgical intervention, administration of MTX, and expectant management. Individualized treatment decisions are crucial and tailored to the specific circumstances of each case [8].

While HP poses inherent risks, the likelihood of recurrence is exceedingly low [8]. This offers reassurance for individuals who have experienced this condition in previous pregnancies. Identified risk factors for HP include a history of pelvic inflammatory disease, previous ectopic pregnancy, prior abdominal and pelvic surgery, or a history of abortion [6]. Recognizing and addressing these risk factors are pivotal components of preventive measures. The utilization of ART, notably in vitro fertilization (IVF), increases the risk of HP. The estimated incidence of pregnancies resulting from ART procedures ranges from one in 100 to one in 500, emphasizing the importance of close monitoring and early intervention in these cases [2]. Prognostically, extrauterine pregnancies within the context of HP are non-viable and pose a significant threat to maternal health. Prompt medical intervention is essential to prevent adverse outcomes, underscoring the critical importance of swift diagnosis and treatment [9].

Historical Context of Fetal Reduction in HP

The historical background of fetal reduction in HP can be traced back to the early 2000s when there were reports of some success with ultrasound-guided fetal reduction using KCl [10]. This procedure involves injecting KCl into the ectopic pregnancy to halt the fetal heartbeat, facilitating the resorption of the ectopic fetus. In 2002, a case report documented the effective management of an HP through selective embryo reduction using KCl [11]. The patient, a 38-year-old primigravida referred to the center at 11+2 weeks, had an ectopic pregnancy situated in the cornual region. Selective cervical fetal reduction was performed through ultrasound-guided intracardiac KCl injection. However, at nine weeks, the patient was readmitted with chorioamnionitis, intrauterine fetal demise, and significant bleeding, leading to the necessity of an abdominal hysterectomy [12].

Another case report from 2012 recounted the successful management of an HP using a combination of KCl and MTX [12]. In this instance, a 38-year-old primigravida presented at 11+2 weeks with an HP located in the cornual region. Similarly, selective cervical fetal reduction was performed using ultrasound-guided intracardiac KCl injection. Subsequently, the patient received MTX to inhibit the growth of the remaining ectopic pregnancy. Remarkably, the IUP continued, resulting in the delivery of a liveborn baby at full term [12]. These early case reports underscored the potential of fetal reduction using KCl in managing HP. However, a cautious approach is necessary to prevent teratogenic effects on viable IUP. Alternatives for treating HP include surgery, MTX, and expectant management, with the choice of treatment tailored to the individual case [10-13].

KCl infusion

Mechanism of Action

KCl, a compound of potassium and chloride ions, is crucial in various physiological processes. Potassium ions are key in maintaining intracellular tonicity, transmitting nerve impulses, and facilitating cardiac, skeletal, and smooth muscle contraction. In the context of medical interventions, KCl infusion has found application in fetal reduction procedures for HP. This involves the injection of KCl directly into the ectopic pregnancy, aiming to cease the fetal heartbeat and induce the resorption of the ectopic fetus. Typically conducted under ultrasound guidance, the procedure entails administering the injection incrementally until cardiac asystole is achieved. Beyond its role in fetal reduction, KCl is also utilized to prevent and treat hypokalemia, which can lead to disruptions in baroreflex sensitivity and electrolyte balance [11,14,15].

Safety Considerations

Caution must be exercised when utilizing KCl infusion, given its potential for serious adverse effects. Direct injection of high concentrations of KCl into a patient can result in instantaneous fatality, necessitating the dilution of KCl for injection concentrate before administration to prevent fatal cardiac arrhythmia and cardiac arrest [16]. The infusion process should be conducted slowly to mitigate the risk of potassium intoxication, and meticulous attention is required to discontinue pumping action before the container is emptied to avoid the occurrence of air embolism [16]. Administration of KCl to pregnant women should only be undertaken when necessary. The lack of animal reproduction studies on KCl raises uncertainties about its potential for causing fetal harm when administered to pregnant women [16]. There is also a notable concern regarding inadvertent miscalculation leading to the administration of higher-than-intended doses of KCl [17]. Regularly monitoring the infusion site for signs of redness and inflammation is imperative. To prevent phlebitis and pain, higher concentrations of KCl should be administered via a central intravenous line [18]. During the infusion procedure, ultrasound guidance is crucial to ensure accurate injection placement, and the injection itself should be delivered in small increments until cardiac asystole is successfully achieved [19]. These precautions underscore the need for meticulous attention and adherence to safety protocols when employing KCl infusion in medical procedures.

Previous Applications in Medical Procedures

Advancements in medical procedures have continually progressed, introducing novel technologies and techniques to enhance patient outcomes. Some of the latest breakthroughs in medical technology encompass mRNA technology, 3D printing, bioprinting, and artificial intelligence [20,21]. Beyond surgical interventions, non-surgical medical procedures are crucial in diagnosing, measuring, monitoring, or treating various conditions, including diseases or injuries that do not necessitate surgery. Non-surgical procedures include physical examinations, tests, X-rays, scans, endoscopy, and therapeutic interventions [22]. In contrast to contemporary medical practices, historical approaches included peculiar methods such as administering tobacco-infused water into the intestines and utilizing cat pianos for patient treatment [23]. The evolution of robotics represents another significant stride in healthcare, where robots are now employed to conduct intricate surgeries and procedures like cancer coronary artery bypass, gallbladder removal, and tumor removal [24]. These advancements underscore the dynamic nature of medical practices, showcasing a continuous commitment to improving patient care through cutting-edge technologies and methodologies.

The Rationale for Using KCl in Fetal Reduction

KCl has emerged as a pivotal tool in managing complex pregnancies, offering several advantages in the context of fetal reduction. Since its introduction in 1988, KCl has gained prominence for its efficacy in inducing fetal demise, particularly in cases such as HP, effectively ceasing the fetal heartbeat and promoting the resorption of the ectopic fetus [25]. Beyond its role in fetal demise, the intracardiac administration of KCl contributes to a reduced risk of hemorrhage, impacting uteroplacental blood flow and proving beneficial in situations where conditions like placenta previa pose a heightened risk of severe bleeding [12]. Notably, studies have substantiated the safety and effectiveness of intracardiac KCl injection, emphasizing its role as a secure method for induced fetal demise; nevertheless, caution is urged to prevent potential teratogenic effects on viable IUP [25]. Incorporating ultrasound guidance in the procedure ensures precise placement of KCl into the fetal heart, minimizing procedural risks and enhancing overall safety [25]. As an alternative to conventional methods like surgery, MTX, and expectant management, the choice of KCl as a therapeutic approach is contingent upon the specifics of each case, accommodating the preferences of both healthcare providers and patients. This underscores the significance of personalized and comprehensive care in navigating the complexities of fetal reduction in diverse clinical scenarios.

Indications for fetal reduction in HP

Identification of High-Risk Cases

Various identified risk factors include pelvic inflammatory disease, a history of ectopic pregnancy, prior abdominal and pelvic surgeries, previous abortion, the use of intrauterine devices, age over 35 years, frequent vaginal douching, smoking, and engagement with ART [6,26]. Notably, the most significant risk factor associated with HP is ART, with women undergoing IVF facing a risk of up to eight times greater [26]. Timely diagnosis is paramount, and transvaginal ultrasound becomes crucial for accurate identification, especially in high-risk cases, such as those involving ART procedures [26]. Patients with HP are at an elevated risk of complications, including tubal rupture, hypovolemic shock, and the potential for maternal or fetal death due to the inherent challenges in diagnosing this condition [26]. Hence, maintaining a high index of suspicion and conducting early, thorough evaluations are imperative, mainly when known risk factors are present.

Medical and Obstetric Criteria

Identifying high-risk cases of HP necessitates a comprehensive understanding of various risk factors and the strategic application of appropriate diagnostic methods [26]. Particularly noteworthy is the heightened risk linked to ART, where women undergoing IVF face an up to eight times greater risk of HP [26]. In ensuring early diagnosis, transvaginal ultrasound emerges as a critical diagnostic tool, especially in cases presenting high risk, such as those involving ART procedures [26]. The complexity of diagnosing HP underscores the elevated risks for patients, including complications like tubal rupture, hypovolemic shock, and the potential for maternal or fetal death [26]. Consequently, maintaining a vigilant and informed approach, marked by a high index of suspicion and early, thorough evaluation, becomes indispensable, mainly when known risk factors are present. This proactive stance is crucial for timely intervention and effective management in cases of HP.

The Decision-Making Process for Recommending a Fetal Reduction

The decision-making process regarding the recommendation of fetal reduction in HP requires meticulous evaluation of the unique aspects of each case and the inherent risks involved. Typically, selective fetal reduction emerges as the primary intervention and is best performed promptly following diagnosis [27]. This procedure is specifically indicated when the ectopic pregnancy poses a substantial risk to either the maternal health or the ongoing IUP [10]. Deciding on fetal reduction is an individualized process, considering the specific risks associated with each case, particularly when the ectopic pregnancy poses a threat to the well-being of the mother or the viability of the IUP [12]. The careful assessment of risks and benefits is crucial in this decision-making process, emphasizing the need to account for the unique circumstances of each case to ensure the most appropriate course of action.

Procedure for fetal reduction

Pre-procedural Assessments

The pre-procedural assessment is a critical phase involving several comprehensive components to ensure a medical intervention's safety and success. Firstly, an exhaustive review of the patient's medical history is conducted, encompassing an examination of pre-existing conditions, previous surgeries, and the current medications the patient is undergoing. This meticulous exploration lays the groundwork for tailoring the upcoming procedure to the individual's unique health profile [28]. Following this, a thorough physical examination is undertaken to assess the patient's overall health and identify any potential risk factors that might impact the planned medical procedure. This hands-on evaluation provides valuable insights into the patient's physiological state, enabling a more informed and personalized approach to the intervention [29]. Additionally, preoperative laboratory studies are initiated depending on the nature of the procedure and the individual patient's circumstances. These may include blood tests, urinalysis, and other relevant investigations, offering a detailed understanding of the patient's current medical condition and aiding in formulating a well-informed procedural strategy [29].

Further imaging studies, such as ultrasound or echocardiography, may be recommended in some instances to gain a more comprehensive understanding of the patient's condition. These studies contribute valuable insights into anatomical considerations and are crucial in refining the procedural approach [29]. Patient counseling is pivotal to the pre-procedural assessment, providing detailed information about the planned procedure. This includes elucidating potential risks, an outline of the expected postoperative course, and addressing any concerns or questions the patient may have. Open communication during this phase is instrumental in securing the patient's informed consent and mental readiness for the upcoming intervention [30]. Lastly, for anesthesia procedures, a preoperative assessment interview with an anesthesiologist is conducted to evaluate the patient's suitability for anesthesia. This involves a focused review of the patient's medical history, physical health, and considerations relevant to administering anesthesia safely [31]. This facet ensures that the patient is well-prepared and physically fit for the specific anesthesia regimen planned for the procedure. In essence, the pre-procedural assessment is a holistic and tailored process, where each component plays a crucial role in ensuring a thorough understanding of the patient's health and needs, ultimately contributing to the overall success and safety of the impending medical intervention. Clear communication and adherence to provided instructions are foundational for achieving a comprehensive and practical pre-procedural assessment.

Administration of KCl

Oral administration: KCl is commonly prescribed, and patients are advised to take it as directed by their healthcare provider. To minimize the risk of stomach upset, it is recommended to ingest each dose with a meal and a full glass of water [32]. Extended-release formulations of KCl are particularly beneficial for individuals who may have difficulty tolerating or adhering to other formulations. However, caution is advised when administering extended-release preparations to patients with conditions that may delay the passage of tablets through the gastrointestinal tract [15].

Intravenous administration of KCl requires careful attention to ensure safety and effectiveness. The preferred method involves slow infusion through a vein, and it should always be diluted in 0.9% sodium chloride to maintain a suitable concentration. It is crucial to avoid exceeding a potassium concentration of 40 mmol/liter in the infusion fluid to prevent potential complications [33]. The rate of intravenous administration should be meticulously controlled to minimize the risk of adverse effects. When administering KCl via a peripheral line, it is essential to avoid overly concentrated solutions, as higher strengths can lead to complications such as phlebitis and pain [18]. This underscores the importance of precise and cautious administration protocols to optimize the therapeutic benefits while minimizing potential risks.

Monitoring and Management During the Procedure

Fetal monitoring: Continuous electronic fetal monitoring plays a pivotal role during fetal reduction procedures, providing real-time assessment of crucial parameters related to fetal well-being. This includes evaluating fetal heart rate (FHR) variability, accelerations, and decelerations and analyzing continuous electronic fetal monitoring patterns over time [34]. This comprehensive monitoring strategy ensures a vigilant observation of fetal responses throughout the procedure, allowing for immediate identification of any deviations from expected patterns and enabling timely interventions if needed.

Intravenous administration: In cases where KCl is employed for fetal reduction, its administration involves a meticulous intravenous infusion. The substance must be slowly administered intravenously, with the added precaution of dilution in 0.9% sodium chloride. The rate of intravenous administration demands careful control, and the potassium concentration for peripheral intravenous administration should be closely monitored to prevent potential complications [19]. This cautious approach emphasizes the significance of precision in drug delivery to optimize therapeutic outcomes while minimizing risks.

Maternal monitoring: Close monitoring of the mother's vital signs and overall condition is paramount throughout fetal reduction. This vigilance ensures the prompt detection of any adverse reactions or complications that may arise during the intervention [35]. Regular assessment of maternal well-being contributes to proactive responses to unexpected developments, fostering a safer procedural environment.

Post-procedural care: Following the fetal reduction procedure, continuous monitoring remains imperative, focusing on both the mother and the remaining fetus(es). This post-procedural care ensures that the ongoing pregnancy progresses without encountering further complications [36]. The sustained vigilance during this period is crucial for identifying and addressing any potential issues that may arise after the procedure, promoting optimal maternal and fetal health outcomes.

Post-procedural Care

The post-procedural care following fetal reduction, also recognized as multifetal pregnancy reduction (MFPR), may entail an overnight hospital stay, specific instructions for maintaining a healthy pregnancy, and the potential need for additional tests and doctor check-ins. While infections arising from the procedure are rare, there exists a slight risk of miscarriage and premature labor. Patients might receive recommendations to adhere to a high-calorie, high-protein diet, take prescribed medication, or undergo additional bed rest to support the remaining fetuses. Counseling and emotional support, including discussions with a counselor, are strongly advised for patients and their partners to facilitate informed decision-making and help cope with the emotional facets associated with the procedure [37]. The American College of Obstetricians and Gynecologists (ACOG) underscores the significance of patient counseling and respecting patients' autonomy in determining whether to proceed with or reduce the number of fetuses. This decision involves carefully considering medical, ethical, religious, and socioeconomic factors, and patients should be equipped with the latest information to make informed choices [38,39]. Regarding the actual procedure, a study on embryo reduction discusses using a transvaginal ultrasound-guided embryo reduction technique, conducted between the seventh and eighth weeks of gestation, reported to be both practical and safe [36]. Another case report outlines the successful non-surgical management of heterotopic abdominal pregnancy following embryo transfer with cryopreserved-thawed embryos. In this instance, ultrasound-guided transvaginal injection of KCl into the abdominal pregnancy resulted in the termination of the ectopic fetus [19]. These advancements underscore the diverse approaches and evolving fetal reduction techniques, emphasizing safety and efficacy in managing complex pregnancies.

Efficacy and outcomes

Success Rates of Fetal Reduction with KCl

Extensive research has investigated the effectiveness of fetal reduction through KCl infusion, particularly in multifetal pregnancies and selective reduction. The evidence consistently demonstrates that intracardiac KCl injection stands out as a highly effective and safe method for inducing fetal demise. A retrospective cohort study reported an impressive success rate of 99.5%, underscoring the reliability of this approach [40]. Notably, using KCl becomes more imperative with advancing gestational age, mainly when the goal is to terminate larger fetuses. Significantly, this method is associated with favorable pregnancy outcomes when applied for fetal reduction in multifetal pregnancies [41]. Moreover, a compelling case report contributes to the growing body of evidence by detailing the successful non-surgical management of a heterotopic abdominal pregnancy. In this case, following embryo transfer with cryopreserved-thawed embryos, ultrasound-guided transvaginal injection of KCl into the abdominal pregnancy resulted in fetal demise and the subsequent spontaneous resorption of the ectopic fetus [19]. These documented instances highlight the versatility and effectiveness of KCl infusion for fetal reduction across diverse clinical scenarios.

Complications and Adverse Effects

The utilization of KCl for fetal reduction carries potential complications and adverse effects. Immediate side effects reported encompass maternal pain, anxiety, and the potential for arrhythmia [42]. A study noted that asystole could be observed within one to two minutes of KCl injection, with immediate post-procedural complications, including maternal pain, leaking, and bleeding, which was conservatively managed [42]. Importantly, it is highlighted that potassium supplementation is unlikely to adversely affect the fetus without inducing maternal toxicity [43]. Furthermore, the application of KCl for fetal reduction is recognized as an effective and safe method for inducing fetal demise [25]. Healthcare providers must diligently weigh the potential risks and benefits associated with KCl infusion for fetal reduction, providing comprehensive patient counseling and managing any ensuing complications judiciously.

Long-Term Outcomes for Both the Reduced and Remaining Fetuses

Long-term outcomes for both the reduced and remaining fetuses after fetal reduction with KCl infusion have been scrutinized across diverse clinical scenarios. In a retrospective cohort study, fetal reduction in dichorionic pregnancies was linked to a fourfold increase in the risk of adverse pregnancy outcomes, encompassing miscarriage, stillbirth, or single intrauterine fetal demise [44]. Notably, while there is an increased relative risk, the absolute risk of adverse outcomes remains low. Another study indicated that MFPR yields improvements in pregnancy outcomes, reducing the risks of preterm delivery, low birth weight, and small gestational age. However, it did not entirely reverse the adverse outcomes associated with multiple pregnancies [45]. Furthermore, a case report delved into the long-term neurodevelopmental consequences following selective feticide in monochorionic pregnancies, revealing a 6.8% incidence of neurodevelopmental impairment among survivors [46]. These collective findings imply that fetal reduction with KCl infusion may entail certain long-term risks and outcomes. Therefore, considering the potential risks and benefits is imperative when contemplating this procedure.

Comparison with alternative approaches

Other Methods of Fetal Reduction

A range of methods exists for fetal reduction, encompassing transabdominal, transcervical, or transvaginal approaches, along with chemical, thermal, radiofrequency, or laser techniques, contingent upon factors such as chorionicity [39]. MFPR is designed to decrease higher-order pregnancies, enhancing perinatal outcomes [41]. In the context of unruptured HP, the suggestion to use KCl infusion for fetal reduction has been made. At the same time, cautionary measures are advised against the use of both potassium and MTX due to their teratogenic effects on viable IUP [47]. The acceptability of these options for the couple is contingent upon their social background [47]. Some advocate delaying fetal reduction until the late first trimester, anticipating a natural reduction of the fetus and allowing for anomaly detection [41]. Distinct from MFPR, selective reduction involves choosing fetuses based on health status [30]. Physicians must be cognizant that state and federal laws can impact the provision of selective reduction [38].

Comparative Analysis of Safety and Efficacy

Several studies have undertaken a comparative analysis of the safety and efficacy of different approaches in managing multifetal pregnancies and HP. One study comparing outcomes of multifetal pregnancies that underwent reduction with KCl to those that did not found that the mean neonatal birthweight was smaller in the KCl group [48]. Another study reported a case of successful non-surgical management of a heterotopic abdominal pregnancy using an ultrasound-guided transvaginal injection of KCl into the abdominal pregnancy, resulting in asystole and ultimately leading to a liveborn vaginal delivery at full term [19]. In the context of HP, a study emphasized the preference for ultrasound-guided reduction over surgery to preserve IUP [49]. Expectant management has also been noted as a strategy to avoid potential complications related to surgery, with the transabdominal sonographic-guided aspiration of ectopic gestational embryos identified as having the best maternal outcome [13]. Furthermore, laparoscopic surgery has been reported as a safe and effective treatment for removing ectopic pregnancy without compromising the IUP [50]. These diverse findings contribute valuable insights into the nuanced considerations in managing multifetal pregnancies and HP, highlighting the importance of tailored approaches based on individual cases.

Considerations for Choosing KCl Infusion

Depending on the specific clinical scenario, KCl dosing is subject to variation. For intravenous administration, the infusion rate should generally not exceed 10-20 mmol per hour, with higher rates potentially necessitating cardiac monitoring [49-51]. It is imperative to administer KCl via a volumetric infusion pump, and regular checks of the infusion site for redness and inflammation are recommended. Care must be exercised to prevent paravenous administration or extravasation, given that concentrated KCl solutions can cause tissue damage. Additionally, potassium concentration for intravenous administration via a peripheral line should not be excessively high, as more substantial concentrations may induce phlebitis and pain [49-51]. Routine monitoring of electrolyte levels is essential to assess the need for further infusions and avoid hyperkalemia and fluid overload. Higher infusion rates may necessitate cardiac monitoring. Patients with or at risk of hyperchloremia should undergo monitoring for plasma chloride levels and renal function [48]. Furthermore, intravenous potassium administration should be reserved for situations where the oral or enteral route is either unavailable or incapable of achieving adequate levels [50,51].

## Conclusions

This comprehensive review has thoroughly examined fetal reduction using KCl infusion in unruptured HP. The key findings underscore the efficacy of this procedure in mitigating the elevated risks associated with heterotopic gestations, emphasizing its potential as a valuable clinical intervention. The safety considerations surrounding KCl infusion and the importance of careful patient selection and counseling have been highlighted. These insights hold significant implications for clinical practice in reproductive medicine. Clinicians are encouraged to consider fetal reduction with KCl in cases where the benefits of reducing the multiple gestations outweigh the associated risks, particularly in scenarios where the risk of rupture poses a substantial threat to maternal and fetal well-being. Recommendations for future studies have been outlined to advance the field further. Ongoing research efforts should concentrate on refining procedural techniques, exploring alternative methods, and assessing the long-term outcomes for both reduced and remaining fetuses. Ethical considerations and the psychosocial impact on patients warrant further investigation to ensure a holistic and patient-centered approach. As this review lays the groundwork for future advancements, it calls for collaborative efforts among researchers and practitioners to address the complexities of managing unruptured HP and continually refine fetal reduction strategies for enhanced clinical outcomes.

## References

[1] L Klitzman R (2016). Reducing the number of fetuses in a pregnancy: providers' and patients' views of challenges. Hum Reprod.

[2] Wang YN, Zheng LW, Fu LL, Xu Y, Zhang XY (2023). Heterotopic pregnancy after assisted reproductive techniques with favorable outcome of the intrauterine pregnancy: a case report. World J Clin Cases.

[3] Nabi U, Yousaf A, Ghaffar F, Sajid S, Ahmed MM (2019). Heterotopic pregnancy - a diagnostic challenge. Six case reports and literature review. Cureus.

[4] Nguyen KP, Hudspeth M, Milestone H (2022). Spontaneous heterotopic pregnancy: diagnosis and management. Case Rep Obstet Gynecol.

[5] Kajdy A, Muzyka-Placzyńska K, Filipecka-Tyczka D, Modzelewski J, Stańczyk M, Rabijewski M (2021). A unique case of diagnosis of a heterotopic pregnancy at 26 weeks - case report and literature review. BMC Pregnancy Childbirth.

[6] Maleki A, Khalid N, Rajesh Patel C, El-Mahdi E (2021). The rising incidence of heterotopic pregnancy: current perspectives and associations with in-vitro fertilization. Eur J Obstet Gynecol Reprod Biol.

[7] Farnaghi S, Kothari A (2013). Heterotopic pregnancy: a report of two cases. Australas J Ultrasound Med.

[8] (2024). Heterotopic Pregnancy. https://www.isuog.org/clinical-resources/patient-information-series/patient-information-pregnancy-conditions/early-pregnancy/heterotopic-pregnancy.html.

[9] Mummert T, Gnugnoli DM (2024). Ectopic Pregnancy. StatPearls [Internet].

[10] Qiong Z, Yanping L, Deep JP, Lin Z (2011). Treatment of cornual heterotopic pregnancy via selective reduction without feticide drug. J Minim Invasive Gynecol.

[11] Wright A, Kowalczyk CL, Quintero R, Leach RE (1996). Selective embryo reduction in a heterotopic pregnancy using potassium chloride injection resulting in a hematosalpinx. Fertil Steril.

[12] Deka D, Bahadur A, Singh A, Malhotra N (2012). Successful management of heterotopic pregnancy after fetal reduction using potassium chloride and methotrexate. J Hum Reprod Sci.

[13] Li JB, Kong LZ, Yang JB, Niu G, Fan L, Huang JZ, Chen SQ (2016). Management of heterotopic pregnancy: experience from 1 tertiary medical center. Medicine (Baltimore).

[14] (2024). Potassium Chloride. https://go.drugbank.com/drugs/DB00761.

[15] Sur M, Mohiuddin SS (2024). Potassium. StatPearls [Internet].

[16] (2024). Potassium Chloride for Injection Concentrate, USP Warnings and Precautions. https://www.pfizermedicalinformation.com/potassium-chloride/warnings.

[17] Nakatani K, Nakagami-Yamaguchi E, Shinoda Y, Tomita S, Nakatani T (2019). Improving the safety of high-concentration potassium chloride injection. BMJ Open Qual.

[18] (2024). How Should Intravenous (IV) Potassium Chloride Be Administered in Adults?. https://www.sps.nhs.uk/articles/how-should-intravenous-iv-potassium-chloride-be-administered-in-adults/.

[19] Scheiber MD, Cedars MI (1999). Successful non-surgical management of a heterotopic abdominal pregnancy following embryo transfer with cryopreserved-thawed embryos. Hum Reprod.

[20] (2024). Top 10 New Medical Technologies 2022. https://www.proclinical.com/blogs/2022-4/top-10-new-medical-technologies-2022.

[21] Basu K, Sinha R, Ong A, Basu T (2020). Artificial intelligence: how is it changing medical sciences and its future?. Indian J Dermatol.

[22] Services D of H& H (2024). Medical Procedures - Non-surgical. http://www.betterhealth.vic.gov.au/health/conditionsandtreatments/medical-procedures-non-surgical.

[23] Beheler T: 10 Strange Medical (2024). 10 Strange Medical Practices from History. https://blogs.loc.gov/headlinesandheroes/2022/04/10-strange-medical-practices-from-history.

[24] (2024). Robotics in Healthcare: Past, present, and future. https://www.ahu.edu/blog/robotics-in-healthcare.

[25] Sfakianaki AK, Davis KJ, Copel JA, Stanwood NL, Lipkind HS (2014). Potassium chloride-induced fetal demise: a retrospective cohort study of efficacy and safety. J Ultrasound Med.

[26] Cucinella G, Gullo G, Etrusco A, Dolce E, Culmone S, Buzzaccarini G (2021). Early diagnosis and surgical management of heterotopic pregnancy allows us to save the intrauterine pregnancy. Prz Menopauzalny.

[27] Wang Y, Niu Z, Tao L, Yang Y, Ma C, Li R (2020). Early intervention for heterotopic caesarean scar pregnancy to preserve intrauterine pregnancy may improve outcomes: a retrospective cohort study. Reprod Biomed Online.

[28] (2024). Pre-procedure Assessment. https://centralflorida.ufhealth.org/patients-visitors/patient-resources/pre-procedure-assessment/.

[29] King MS (2000). Preoperative evaluation. Am Fam Physician.

[30] (2024). Multifetal Pregnancy Reduction. https://www.acog.org/clinical/clinical-guidance/committee-opinion/articles/2017/09/multifetal-pregnancy-reduction.

[31] (2024). Pre-procedural Assessment at Inova Fairfax Medical Campus. https://www.inova.org/locations/pre-surgical-services.

[32] (2024). Potassium Chloride (Rx). https://reference.medscape.com/drug/kdur-slow-k-potassium-chloride-344450.

[33] POTASSIUM CHLORIDE 10% = KCl 10 (2024). Potassium Chloride 10% = KCl 10% Injectable. https://medicalguidelines.msf.org/en/viewport/EssDr/english/potassium-chloride-10-kcl-10-injectable-16683070.html.

[34] Arnold JJ, Gawrys BL (2020). Intrapartum fetal monitoring. Am Fam Physician.

[35] Dalal AR, Purandare AC (2018). The partograph in childbirth: an absolute essentiality or a mere exercise?. J Obstet Gynaecol India.

[36] Talwar P, Sharma RK, Sandeep K, Sareen S, Duggal BS (2011). Embryo reduction: our experience. Med J Armed Forces India.

[37] (2024). What Is Multifetal Reduction?. https://www.webmd.com/infertility-and-reproduction/fertility-multifetal-reduction.

[38] Beriwal S, Impey L, Ioannou C (2020). Multifetal pregnancy reduction and selective termination. Obstet Gynecol.

[39] Govender L, Moodley J (2012). Late termination of pregnancy by intracardiac potassium chloride injection: 5 years' experience at a tertiary referral centre. S Afr Med J.

[40] Lee JR, Ku SY, Jee BC, Suh CS, Kim KC, Kim SH (2008). Pregnancy outcomes of different methods for multifetal pregnancy reduction: a comparative study. J Korean Med Sci.

[41] Dey M, Saraswat M (2018). Outcomes of multifetal reduction: a hospital-based study. J Obstet Gynaecol India.

[42] Gomes CF, Sousa M, Lourenço I, Martins D, Torres J (2018). Gastrointestinal diseases during pregnancy: what does the gastroenterologist need to know?. Ann Gastroenterol.

[43] Kristensen SE, Ekelund CK, Sandager P (2023). Risks and pregnancy outcome after fetal reduction in dichorionic twin pregnancies: a Danish national retrospective cohort study. Am J Obstet Gynecol.

[44] Yimin Z, Minyue T, Yanling F (2022). Fetal reduction could improve but not completely reverse the pregnancy outcomes of multiple pregnancies: experience from a single center. Front Endocrinol (Lausanne).

[45] van Klink J, Koopman HM, Middeldorp JM, Klumper FJ, Rijken M, Oepkes D, Lopriore E (2015). Long-term neurodevelopmental outcome after selective feticide in monochorionic pregnancies. BJOG.

[46] Dodd JM, Dowswell T, Crowther CA (2015). Reduction of the number of fetuses for women with a multiple pregnancy. Cochrane Database Syst Rev.

[47] Zhou AJ, Li L, Wang HM (2019). Comparisons between two methods of multifetal pregnancy reduction in women with a dichorionic triamniotic triplet pregnancy. Taiwan J Obstet Gynecol.

[48] Patel PH, Zulfiqar H (2024). Reverse Transcriptase Inhibitors. StatPearls [Internet].

[49] Ge F, Ding W, Zhao K, Qu P (2023). Management of heterotopic pregnancy: clinical analysis of sixty-five cases from a single institution. Front Med (Lausanne).

[50] Chen S, Zhu Y, Xie M (2022). Comparison of laparoscopic and open approach in the treatment of heterotopic pregnancy following embryo transfer. Front Surg.

[51] McMahon RS, Bashir K (2024). Potassium Chloride. StatPearls [Internet].

